# The RNA-Binding Protein BoRHON1 Positively Regulates the Accumulation of Aliphatic Glucosinolates in Cabbage

**DOI:** 10.3390/ijms25105314

**Published:** 2024-05-13

**Authors:** Xue Bai, Ruixing Zhang, Qi Zeng, Wenjing Yang, Fang Fang, Qingguo Sun, Chengtai Yan, Fangguan Li, Xifan Liu, Baohua Li

**Affiliations:** State Key Laboratory of Crop Stress Biology for Arid Area, College of Horticulture, Northwest A&F University, Yangling, Xianyang 712100, China; xue.bai@nwafu.edu.cn (X.B.); zrxnihao@163.com (R.Z.); zengqi@nwafu.edu.cn (Q.Z.); yangwenjing@nwafu.edu.cn (W.Y.); 2022205044@mail.nwpu.edu.cn (F.F.); qguosun@nwafu.edu.cn (Q.S.); yan1618226@nwafu.edu.cn (C.Y.); 18364798737@163.com (F.L.); liuxifan2020051572@nwafu.edu.cn (X.L.)

**Keywords:** glucosinolates, cabbage, BoMYB28, BoRHON1

## Abstract

Aliphatic glucosinolates are an abundant group of plant secondary metabolites in Brassica vegetables, with some of their degradation products demonstrating significant anti-cancer effects. The transcription factors MYB28 and MYB29 play key roles in the transcriptional regulation of aliphatic glucosinolates biosynthesis, but little is known about whether BoMYB28 and BoMYB29 are also modulated by upstream regulators or how, nor their gene regulatory networks. In this study, we first explored the hierarchical transcriptional regulatory networks of MYB28 and MYB29 in a model plant, then systemically screened the regulators of the three *BoMYB28* homologs in cabbage using a yeast one-hybrid. Furthermore, we selected a novel RNA binding protein, BoRHON1, to functionally validate its roles in modulating aliphatic glucosinolates biosynthesis. Importantly, BoRHON1 induced the accumulation of all detectable aliphatic and indolic glucosinolates, and the net photosynthetic rates of *BoRHON1* overexpression lines were significantly increased. Interestingly, the growth and biomass of these overexpression lines of *BoRHON1* remained the same as those of the control plants. BoRHON1 was shown to be a novel, potent, positive regulator of glucosinolates biosynthesis, as well as a novel regulator of normal plant growth and development, while significantly increasing plants’ defense costs.

## 1. Introduction

The huge numbers of highly structurally and functionally diverse metabolites that plants produce can be roughly divided into plant primary metabolites, plant secondary metabolites, and plant hormones [1]. Among these, plant secondary metabolites make up the majority, also known as plant specialized metabolites [2,3,4]. They are often lineage-specific and determine plants’ interactions with their surrounding organisms and environments. Importantly, many of them are natural nutrients with pharmaceutical functions [5,6,7,8].

For example, glucosinolates are plentiful plant secondary metabolites uniquely present in Brassicales, including the model plant Arabidopsis and Brassica vegetables [9]. Some of the hydrolysis products of glucosinolates are key nutrients in the human diet; sulforaphane, the isothiocyanate hydrolysis of the aliphatic glucosinolate glucoraphanin, is a highly valuable anticancer metabolite that has been intensively researched and investigated in recent decades [10,11]. Increasing the accumulation of beneficial glucosinolates and optimizing their dietary benefits remain major concerns for plant biologists, as well as for researchers in nutrition, medical workers, and the general public [7,12,13].

Owing to the fact that the model plant Arabidopsis belongs to Brassicales [14], glucosinolates have been intensively studied and have gradually evolved as crucial model systems for researching plants’ secondary metabolism [6]. Much of our understanding of biosynthetic glucosinolate pathways was elucidated around 15 years ago [9,15], and the regulatory mechanisms of glucosinolates pathways, as well as plants’ secondary metabolisms pathways in general, have been pivotal in this research field [16,17]. MYB28 and MYB29 were found to play key roles in the transcriptional regulation of aliphatic glucosinolates biosynthesis; together, they operate as an effective and powerful molecular switch for activating and deactivating the aliphatic glucosinolates pathway [18,19,20]. After the identification of MYB28 and MYB29 as core transcriptional regulators in the aliphatic glucosinolates pathway, similar core regulators were also uncovered in multiple plant secondary metabolism pathways. These include MYB34, MYB51, and MYB122 in the indolic glucosinolates pathway [21,22,23,24,25]; MYC2, MYC3, and MYC4 in all types of glucosinolates pathways [26]; Bl (Bitter leaf) and Bt (Bitter fruit) for the production of cucurbitacin C in cucumbers [27]; bHLH2 in the amygdalin biosynthetic pathway in almonds [28]; and LMI1 in the production of essential oil in citrus [29]. In light of these exciting advancements, the high translational potential of transcriptional regulators of plants’ secondary metabolism pathways has sparked substantial research interest and further study.

We successfully identified and validated dozens of novel regulators of biosynthetic genes in the aliphatic glucosinolate pathway in the model plant Arabidopsis [13,30,31,32,33]. In this current study, we further explored the upstream regulators of *BoMYB28s* in the aliphatic glucosinolates pathway in cabbage, not only since cabbage is a staple vegetable around the world but also since rich genomic resources and research database information exists for this type of plant [34,35]. We screened and identified the first regulatory networks for *BoMYB28s* through a yeast one-hybrid and selected a novel RNA-binding protein, BoRHON1, for functional validation. *BoRHON1* strongly increased the accumulation of all detectable glucosinolates, both aliphatic and indolic glucosinolates, in the *BoRHON1* overexpression plants. Furthermore, BoRHON1 increased the photosynthetic rates to specifically boost glucosinolates synthesis while maintaining normal plant growth and development.

## 2. Results

### 2.1. Transcriptional Regulatory Networks of MYB28 and MYB29 in Arabidopsis

**Figure 1:**
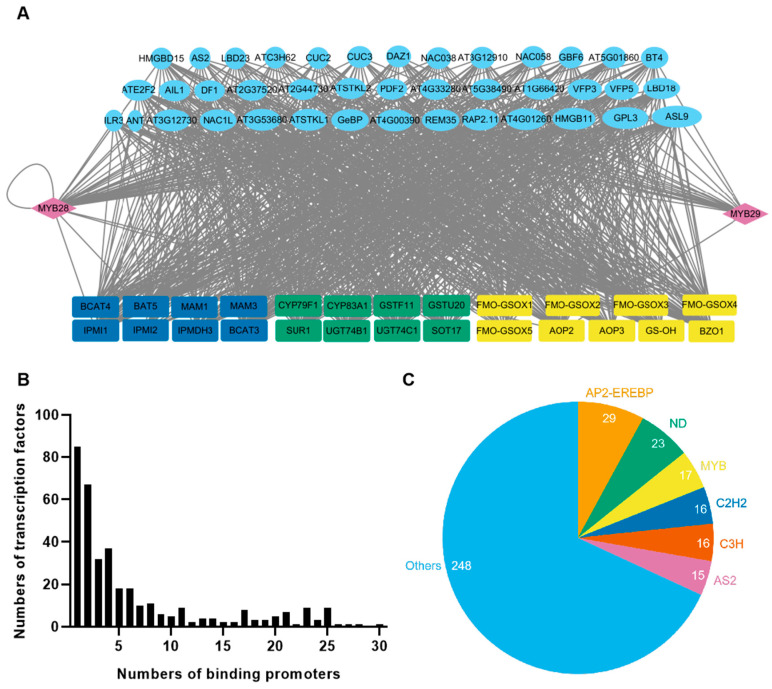
Summary of the regulatory networks of MYB28 and MYB29 in Arabidopsis. (**A**) The interaction networks between transcription factors and the promoters of biosynthetic and regulatory genes using yeast one-hybrid. Transcription factors are shown as blue ellipses, and those binding 20 or more promoters were selected. Purple diamonds indicate genes present as both transcription factors and promoters. Rectangles represent promoter genes. Aliphatic glucosinolates biosynthetic genes are represented in different colors: dark blue for side chain extension pathway genes, green for core structure formation pathway genes, and yellow for side chain modification pathway genes. (**B**) Distribution of the number of transcription factors binding to the promoters of MYB28 and MYB29. (**C**) Distribution of gene families of transcription factors binding to the promoters of MYB28 and MYB29.

We have explored the transcriptional regulation of the aliphatic glucosinolates pathway and successfully identified dozens of new transcriptional regulators of this pathway in the model plant Arabidopsis [30,31]. Although most of the promoters in our yeast one-hybrid screening assay were cloned from biosynthetic genes in the aliphatic glucosinolate pathway, we also cloned the promoters of the core regulators of the aliphatic glucosinolate pathway, MYB28 and MYB29, and screened upstream regulators of MYB28 and MYB29 [13,30]. In total, 2116 interactions were identified, with 364 unique transcription factors binding the promoters of MYB28 and MYB29 (Table S1). Surprisingly, 237 transcription factors could bind the promoter of MYB28, the major regulator of the aliphatic glucosinolate pathway, roughly equaling around 10% of all transcription factors encoded in the Arabidopsis genome. Most of these bind the promoter of MYB28 alongside promoters of the biosynthesis genes in the aliphatic glucosinolate pathway. To visualize the regulatory network, in Cytoscape [36], we selected upstream regulators of MYB28 and MYB29 that could bind 20 or more promoters of the aliphatic glucosinolates genes. Patterns of hierarchical transcriptional regulation clearly emerged (Figure 1A). For example, HMGBD15 binds the promoter of MYB28 as well as *BCAT4*, *BAT5*, *MAM1*, *MAM3*, *CYP79F1*, *CYP83A1*, *GSTF11*, *SUR1*, *UGT74C1*, *SOT17*, *FMO-GSOX1-4*, *AOP2*, *GS-OH*, and *BZO1* (Figure S1). Among the 364 upstream regulators of MYB28 and MYB29, most of them bind 1 to 6 promoters of the biosynthetic genes in the aliphatic glucosinolates pathway from diverse transcriptional factor families (Figure 1B; Table S2), including AP2-EREBP (29), ND (23), MYB (17), C2H2 (16), C3H (16), and AS2 (15) (Figure 1C). This indicates the potential complexity of the transcriptional regulatory networks and mechanisms of the aliphatic glucosinolates pathway, with core regulators MYB28 and MYB29 connecting these regulatory networks.

### 2.2. Identification of Upstream Regulators of BoMYB28s in Cabbage

**Figure 2:**
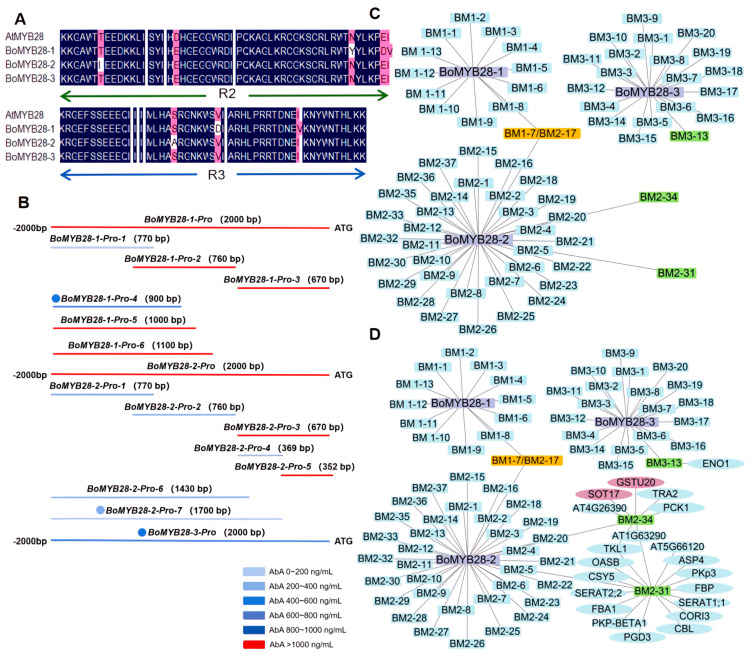
The upstream regulators of *BoMYB28* homologous genes were screened using yeast one-hybrid assay. (**A**) Multiple comparisons of amino acid sequences of BoMYB28 homologous genes. Identical amino acids are indicated by white letters on a black background. (**B**) Screening of optimal promoter regions of *BoMYB28* homologs as baits for yeast one-hybrid assay. (**C**) Regulatory networks of *BoMYB28* homologous genes. (**D**) Association analysis of aliphatic glucosinolates regulatory networks in cabbage and *Arabidopsis thaliana*. In (**C**,**D**), the purple rectangles represent *BoMYB28* homologous genes; yellow, green, and light blue rectangles represent upstream regulated genes selected using yeast one-hybrid assay. Ellipses represent genes in Arabidopsis, and reddish-purple represents aliphatic glucosinolates biosynthetic genes.

Since MYB28 is the major regulator of aliphatic glucosinolates biosynthesis [18,19,20] and MYB28 may be regulated by many upstream regulators in Arabidopsis, in this study, we focused on exploring the upstream regulators of *BoMYB28s* in *Brassica oleracea* var. *capitata* L. (cabbage), as we deemed this pertinent to both Brassica vegetable breeding and diverse translational applications [37]. The *AtMYB28* homologs in Brassicaceae were retrieved from Syntenic Gene@Subgenomes in the Brassicaceae Database (BRAD, http://brassicadb.cn/#/, accessed on 6 March 2021). The results showed that there were three *BoMYB28* genes in cabbage. Their gene numbers were BolC07g043460.2J, BolC02G0604400.2J, and BolC09g007690.2J, named *BoMYB28-1*, *BoMYB28-2*, and *BoMYB28-3*, respectively. Multiple sequence alignment revealed that BoMYB28s contain R2 and R3 domains and are R2R3-MYB members (Figure 2A). The 2000 bp promoter regions upstream of the start codon of all three homologous genes of *BoMYB28s* in cabbage were cloned and tested in terms of the AbA (Aureobasidin A) auto-activation concentration and the screening conditions for the yeast one-hybrid assay. AbA screening greatly eliminates background cloning and is beneficial to the growth and recognition of positive clones. AbA could not inhibit the autoactivation of the *BoMYB28-1* and *BoMYB28-2* yeast strains, possibly because of the dense cis-acting elements attached to their transcription start sites. We further cloned multiple truncated forms of the promoters of *BoMYB28-1* and *BoMYB28-2* and tested for lower auto-activation. Our aim was to explore the regulatory factors upstream of *BoMYB28s*, so we preferentially selected the longest promoter fragment for screening when the AbA concentration could be inhibited. *BoMYB28-1-Pro-4* and *BoMYB28-2-Pro-7*, together with promoter of *BoMYB28-3* which were auto-activated at a low concentration, were selected and used as baits for the following yeast one-hybrid screening (Figure 2B; Table S3). Using a high-quality cabbage cDNA library (Table S4) developed in our lab, we identified 13, 41, and 24 interacting clones for *BoMYB28-1*, *BoMYB28-2*, and *BoMYB28-3*, respectively, and 13, 37, and 20 unique interacting regulators were confirmed using sequencing and BRAD’s Basic Local Alignment Search Tool (BLAST) (http://brassicadb.cn/#/BLAST/, accessed on 28 October 2021) (Figure 2C; Table S5). Interestingly, three upstream regulators, BM2-31, BM2-34, and BM3-13, were shared by Arabidopsis and the current study results (Figure 2D), indicating the connections between the model plant Arabidopsis and Brassica vegetables. These findings constitute the first systemic screening of the upstream regulators of *MYB28* in Brassica vegetables and may point to valuable candidate breeding genes.

### 2.3. BoRHON1 as a Novel Candidate Regulator of the Aliphatic Glucosinolates Pathway for Functional Validation in Cabbage

**Figure 3:**
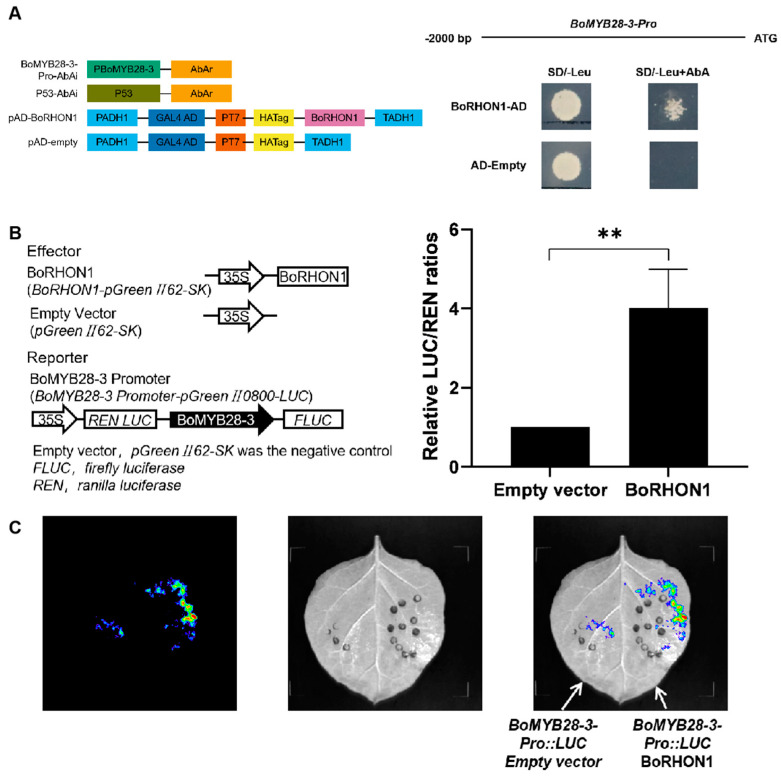
BoRHON1 binds the promoter of *BoMYB28-3*. (**A**) Y1H assay showed that BoRHON1 could bind directly to the *BoMYB28-3* promoter. pGADT7 served as a negative control. (**B**) Dual-luciferase (LUC) assays showed that the BoRHON1 increased *BoMYB28-3* promoter activity. The promoter activity is expressed as the LUC/REN ratio. (**C**) Tobacco transient expression assays showing that BoRHON1 trans-activated the expressions of *BoMYB28-3*. Representative images of *N. benthamiana* leaves 72 h after infiltration are shown. Each piece of data represents the mean of three independent biological replicates (mean ± SE). ** indicates significant differences compared with the control at a *p* value ≤ 0.01.

Among the newly identified upstream regulators, we decided to select the upstream regulators of *BoMYB28-3* for functional validation in this study since they showed novel features. Importantly, of all 24 candidate upstream regulators of *BoMYB28-3*, BolC05g004380.2J, the homolog of RHON1 in Arabidopsis [38,39,40] was independently identified three times in our yeast one-hybrid assay (Table S5). We named it BoRHON1 in the following study results. BoRHON1 could bind the promoter of *BoMYB28-3* in both the yeast one-hybrid assay (Figure 3A) and dual-luciferase assay and thus induce the expression of *BoMYB28-3* (Figure 3B,C). Taken together, these results indicate that BoRHON1 could bind the promoter of *BoMYB28-3* and serve as a novel candidate positive regulator of the aliphatic glucosinolates pathway in cabbage.

### 2.4. Sequence Analyses of BoRHON1

**Figure 4:**
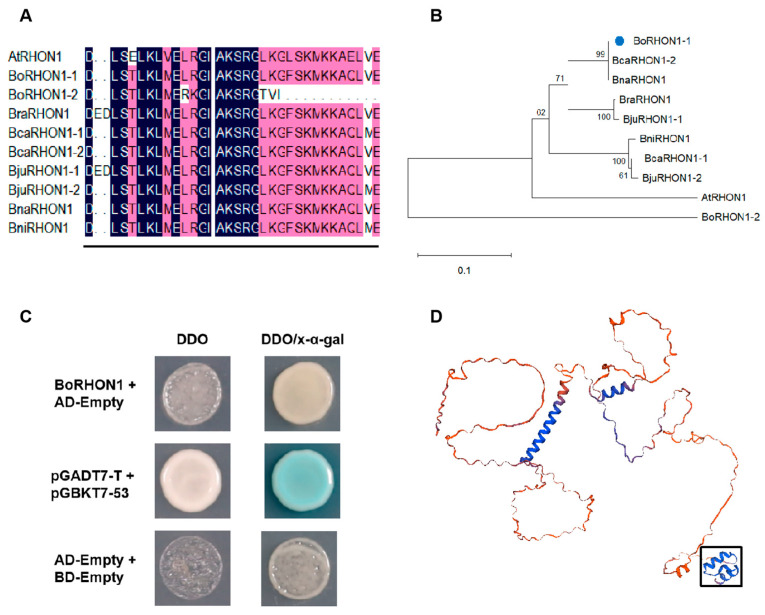
Gene and molecular characteristics of BoRHON1. (**A**) Multiple sequence alignments of RHON1’s amino acid sequences. Identical amino acids are indicated by white letters on a black background. The Rho_N motif is underlined. (**B**) RHON1 phylogenetic tree. Bootstrap values are indicated at each branch node. The blue point represents BoRHON1 (BoRHON1-1). Scale bar indicates the similarity coefficient. (**C**) Detection of transcriptional activation activity of BoRHON1. pGBKT7 empty vector acted as the negative control. (**D**) The putative crystallized structure of BoRHON1. The region demarcated by the black box is the Rho_N motif.

The coding region of *BoRHON1* (BolC05g004380.2J) was cloned from cabbage and shown to encode a protein containing 384 amino acids. The amino acid sequence of BoRHON1 was aligned with the amino acid sequences of homologous genes from multiple Brassica species (*B. rapa*, *B. carinata*, *B. juncea*, *B. napus*, and *B. nigra*) and Arabidopsis. The RHON1s were highly conserved in the different species, and the C-terminal contained the Rho RNA binding domain (Figure 4A). To better understand the evolutionary relationship between BoRHON1 and other RHON1 proteins, we constructed a phylogenetic tree of 10 RHON1 proteins using the neighbor-joining (NJ) method in MEGA X. The phylogenetic analysis revealed a significant evolutionary correlation between BoRHON1 and its homologs in the six other species, while the protein sequence of BoRHON1 had the highest homology with BcaRHON1-2 (BcaC05g24297) and BnaRHON1 (BNC05G0689690-1) (99%) (Figure 4B). Furthermore, transcriptional activation analysis revealed that only the positive control clones could grow normally on a synthetic medium containing X-α-gal (5-bromo-4-chloro-3-indolyl-alpha-D-pyranogalactoside) deficient in tryptophan, leucine, histidine, and adenine (SD/-Trp-Leu-His-Ade). This result indicates that BoRHON1 has no transcriptional activation activity (Figure 4C). Predicting the crystallized structure of BoRHON1 showed the Rho_N motif at the C-terminal (Figure 4D). These results indicate that *BoRHON1* and *AtRHON1* have similar gene structures, and their gene functions may be conserved.

### 2.5. Expression Patterns and Sub-Cellular Localization of BoRHON1

**Figure 5:**
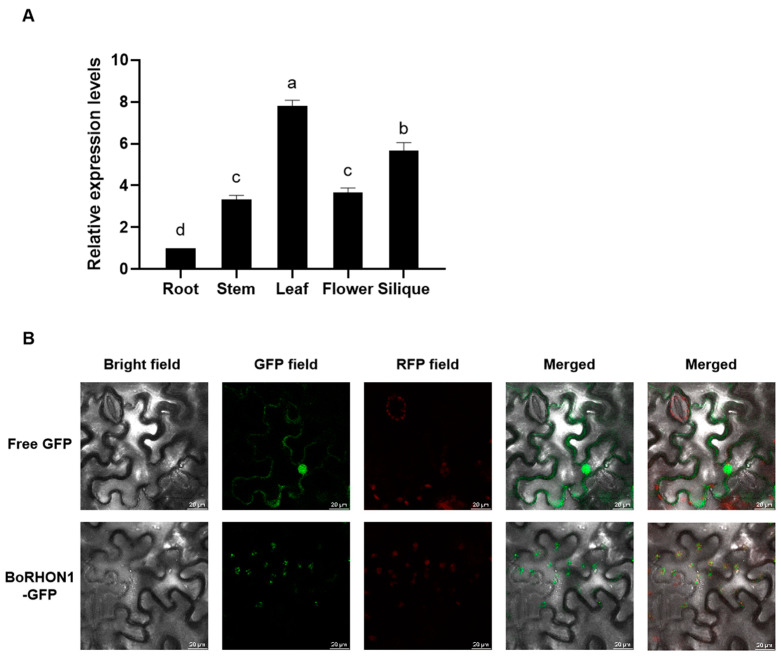
Expression patterns and sub-cellular localization of BoRHON1. (**A**) Transcript levels of *BoRHON1* in different cabbage tissues. Each piece of data represents the mean of three independent biological replicates (mean ± SE). Different letters indicate significant differences according to Tukey’s test (*p* < 0.05). (**B**) The sub-cellular localization of BoRHON1 in *N. benthamiana*. Scale bar = 20 μm.

*BoRHON1* was expressed in all the tested cabbage tissues, including the roots, stems, leaves, flowers, and siliques, while its expression was highest in the leaves (Figure 5A). To further determine the subcellular localization of BoRHON1, the fusion construction of *pGreen-35S:BoRHON1-GFP* and the empty vector *pGreen-35S-GFP* were transformed into tobacco leaves, respectively, and the BoRHON1-GFP fusion protein was located in the chloroplasts (Figure 5B). The leaves are one of the major tissues for glucosinolates biosynthesis, and some of the most energy-intensive steps of glucosinolates biosynthesis take place in the chloroplasts; therefore, the expression patterns and sub-cellular localization of BoRHON1 support its potential roles in modulating glucosinolate biosynthesis.

### 2.6. BoRHON1 Could Induce the Biosynthesis of Both Aliphatic and Indolic Glucosinolates

**Figure 6:**
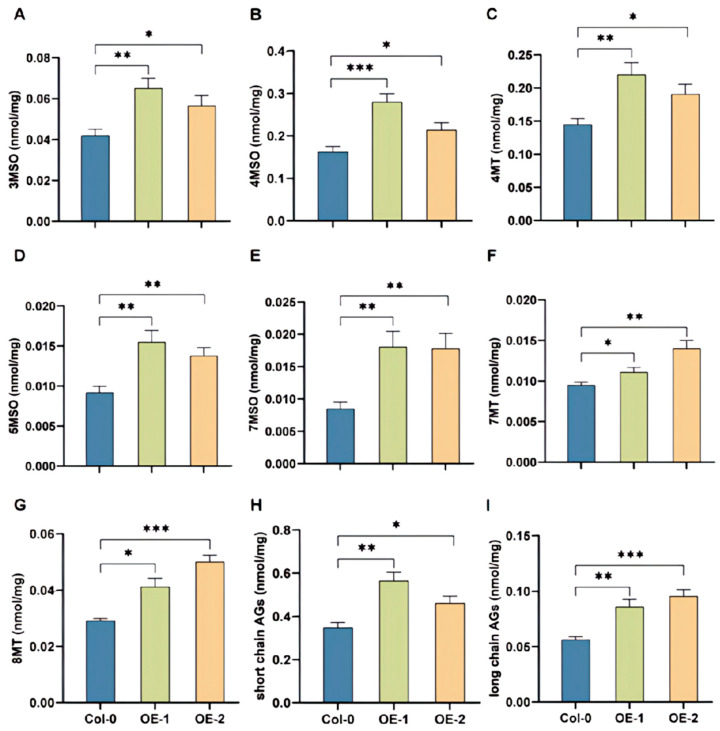
Aliphatic glucosinolates contents in *BoRHON1* overexpression lines. (**A**) 3MSO. (**B**) 4MSO. (**C**) 4MT. (**D**) 5MSO. (**E**) 7MSO. (**F**) 7MT. (**G**) 8MT. (**H**) Short-chain AGs. (**I**) Long-chain AGs. Each piece of data represents the mean of six independent biological replicates (mean ± SE). Student’s *t*-test was adopted to calculate statistical significance (* ≤ 0.05; ** ≤ 0.01; *** ≤ 0.001).

**Figure 7:**
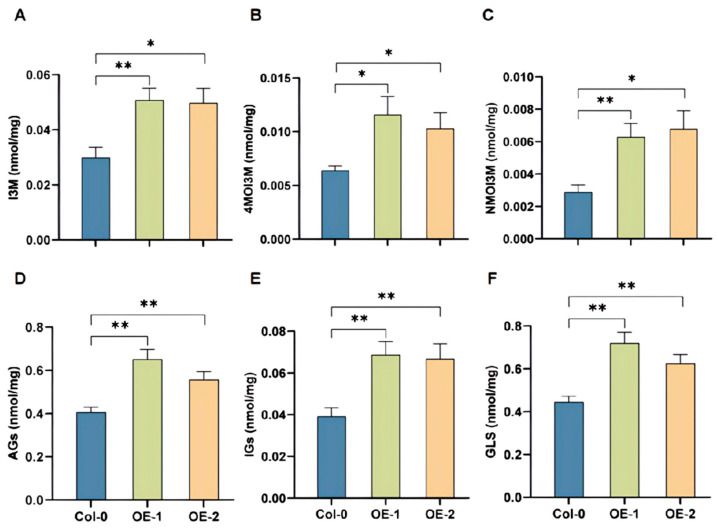
Changes in the content of glucosinolates in each component of *BoRHON1* overexpression lines. (**A**) I3M. (**B**) 4MOI3M. (**C**) NMOI3M. (**D**) AGs. (**E**) IGs. (**F**) GLS. Each piece of data represents the mean of six independent biological replicates (mean ± SE). Student’s *t*-test was adopted to calculate statistical significance (* ≤ 0.05; ** ≤ 0.01).

As the glucosinolates pathway was conserved among the model plant and the Brassica vegetables, *BoRHON1* was overexpressed in Arabidopsis to test its roles in modulating glucosinolates biosynthesis. Two independent homozygous lines of *BoRHON1* were generated and molecularly validated as overexpression lines (Figure S2). Importantly, all detectable aliphatic glucosinolates were induced in the two overexpression lines, including 3MSO (3-methylsulphinylpropyl-GS, glucoiberin) (Figure 6A), 4MSO (4-methylsulphinylbutyl-GS, glucoraphanin) (Figure 6B), 4MT (4-methylthiobutyl-GS, glucoerucin) (Figure 6C), 5MSO (5-methylsulphinylpentyl-GS, glucoalyssin) (Figure 6D), 7MSO (7-methylsulphinylheptyl-GS, glucosiberin) (Figure 6E), 7MT (7-methylthioheptyl-GS) (Figure 6F), and 8MT (8-methylthiooctyl-GS) (Figure 6G), increasing the accumulation of short-chain aliphatic glucosinolates (Figure 6H), long-chain aliphatic glucosinolates (Figure 6I), total aliphatic glucosinolates (Figure 7D), and total glucosinolates (Figure 7F).

Surprisingly, all detectable indolic glucosinolates, including I3M (Indol-3-yl-methyl-GS, glucobrassicin) (Figure 7A), 4MOI3M (4-methoxyindol-3-yl-methyl-GS, 4-meyhoxy glucobrasicin) (Figure 7B), NMOI3M (Figure 7C), and total indolic glucosinolates (Figure 7E), were induced in the two overexpression lines.

As glucosinolate biosynthesis is an energy-intensive process and is predicted to cost around 15% of the photosynthetic energy of plants [41], approximately doubling of all detectable glucosinolates comes at a huge cost for plants and represents a marked phenotype for the biosynthesis of these specialized metabolites, supporting that *BoRHON1* is a strong positive regulator of the glucosinolate pathways in cabbage.

### 2.7. BoRHON1 Increased the Photosynthesis Rate

**Figure 8:**
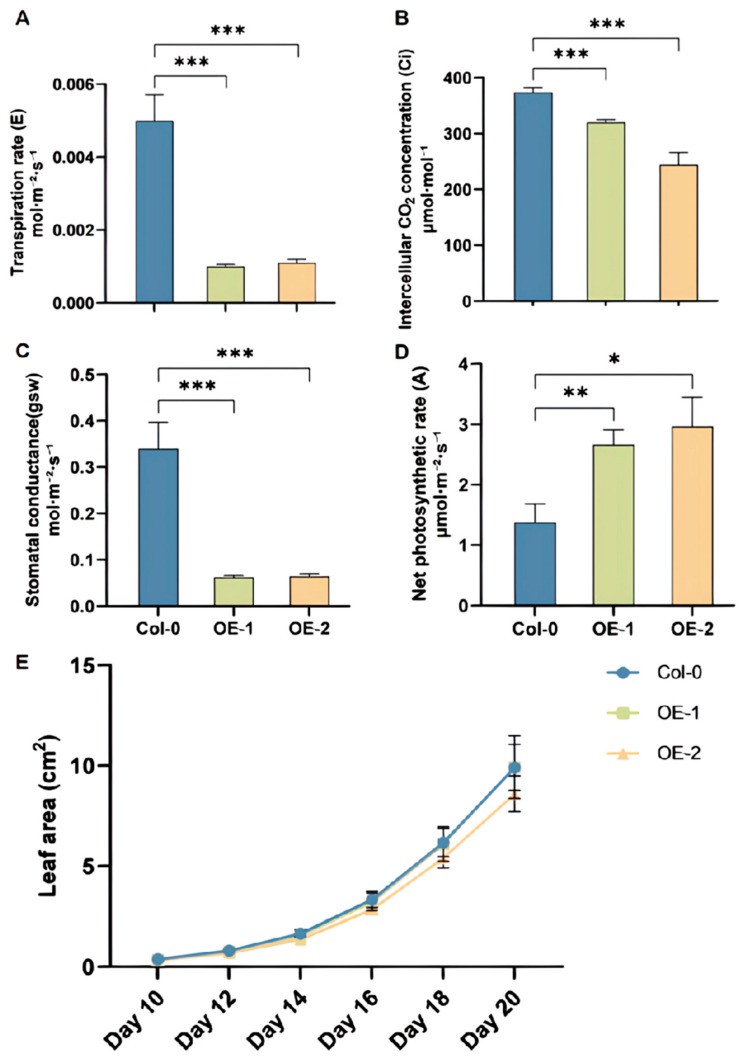
Photosynthesis and growth phenotypes in *BoRHON1* overexpression lines. (**A**) Transpiration rate (E). (**B**) Intercellular CO_2_ concentration (Ci). (**C**) Stomatal conductance (gsw). (**D**) Net photosynthetic rate (A). (**E**) Leaf area of overexpression lines and wild type at each growth stage. In (**A**–**D**), each piece of data represents the mean of three independent biological replicates (mean ± SE). Student’s *t*-test was adopted to calculate statistical significance (* ≤ 0.05; ** ≤ 0.01; *** ≤ 0.001).

The *rhon1* mutant exhibited small leaves, pale green leaves, and albino phenotypes [38,40]. In addition, the Fv/Fm ratio, a chlorophyll fluorescence parameter, was lower in the mutant compared to the wild type, indicating a lower photosystem II efficiency [39,40]. The net photosynthetic rate (A), transpiration rate (E), intercellular CO_2_ concentration (Ci), and stomatal conductance (gsw) of the two overexpression lines were measured. The results showed that the net photosynthetic rates (Figure 8D) of the two overexpression lines were significantly higher than those of the wild type. Meanwhile, the overexpression lines’ transpiration rate (Figure 8A), intercellular CO_2_ concentration (Figure 8B), and stomatal conductance (Figure 8C) were significantly lower than those of the wild type. Due to the significant increase in the net photosynthetic rate in the overexpression lines, we measured the growth and development of the plants according to the leaf area (Figure 8E), plant weight, and flowering time. Crucially, the growth and development of the overexpression plants were not significantly affected (Figures S3–S6). Therefore, we can postulate that the increased energy consumed in photosynthesis in the overexpression lines might have specifically pertained to glucosinolate accumulation while maintaining normal plant growth and development.

## 3. Discussion

### 3.1. The Gene-Regulatory Networks of Plant-Specialized Metabolisms

The identification of the core transcriptional regulators MYB28 and MYB29 in Brassicales was a major advancement in our understanding of the regulatory mechanisms of plants’ secondary metabolism [18,19,20]. As novel core regulators are discovered in multiple horticultural plants [27,28,29], one important question remains: what are the gene-regulatory networks of aliphatic glucosinolates? As the core regulators function as an on/off switch for specialized metabolisms in plants, these transcriptional regulatory mechanisms appear to be straightforward. However, the actual mechanisms have been characterized as much more complex and dynamic [42].

Recently, we conducted a comprehensive analysis of all the aliphatic glucosinolates pathway regulators in Arabidopsis and selected more than 150 new regulators for functional verification. We could show the following: (1) The promoter of MYB28 had the largest number of interacting upstream regulators among all the tested genes; (2) The newly validated regulators of the aliphatic glucosinolate pathways belonged to diverse TF families; (3) The newly validated regulators modulate and coordinate glucosinolate biosynthesis and multiple biological processes. In this current study, we found that the three *BoMYB28s* in cabbage also have large numbers of candidate regulators binding their promoters, supporting the concept that the core regulators MYB28s themselves are also regulated. As a result, these newly identified candidate regulators in cabbage could serve as valuable target genes for breeding new, more nutritious cabbage varieties.

### 3.2. The Regulatory Roles of BoRHON1 in Modulating Glucosinolate Biosynthesis

Before the identification and functional validation of BoRHON1 in this current study, almost all the identified regulators of the aliphatic glucosinolates pathway [13,30], as well as plant secondary metabolite pathways in general, have been transcription factors. As we screened the regulators of the promoters of *BoMYB28* in cabbage, we identified three independent clones, all sequenced and confirmed as BoRHON1, which is an RNA-binding protein [38,39,40]. We decided to explore this novel protein’s roles in regulating aliphatic glucosinolates. Surprisingly, BoRHON1 was characterized as a very strong positive regulator with unprecedented regulatory features: (1) BoRHON1 could induce the accumulation of all detectable glucosinolates, including both aliphatic and indolic glucosinolates; (2) The individual glucosinolates were induced almost evenly, with around 1.5–2 fold as many as those in the control. These remarkable features indicate that BoRHON1 might represent a novel type of regulator with unknown regulatory mechanisms. As the most energy-intensive steps in glucosinolates biosynthesis happened in the chloroplasts, and BoRHON1 is mainly localized in the chloroplasts, we speculated that BoRHON1 augments glucosinolates biosynthesis partly because of its localization in the major energy factory in the plant cells [9,41]. We also demonstrated that BoRHON1 increased the expression of biosynthetic and regulatory genes in the glucosinolate pathway in its overexpression lines, including *MAM3*, *GSOX2*, *GSOX4*, *CYP79B3*, and *MYB34* (Figure S7). This represents a promising direction for future research, furthering exploration into how BoRHON1 positively regulates glucosinolate biosynthesis.

### 3.3. The Interplay between Plant Growth and Defenses

In plants, chloroplasts drive all life-related activities by converting solar energy into energy. When the *rhon1* mutant was grown in soil, its leaves showed an albinism phenotype and could only survive for a few weeks. Further analysis showed that electron transport in PSII was restricted in the mutant, and its Fv/Fm value was significantly reduced compared with that in the wild type [38]. In the current study, we were able to show that the net photosynthetic rate in rosette leaves from the two *BoRHON1* overexpression lines was significantly higher than that in the wild type, indicating that the overexpression of *BoRHON1* could improve plant photosynthesis. In order to investigate whether BoRHON1 could affect plants’ development, we measured the biomass and flowering time of the *BoRHON1* overexpression lines. Interestingly, the growth and flowering time of the overexpression lines remained the same as those of the wild-type control (Figures S4 and S5).

Glucosinolate biosynthesis is extremely energy-intensive, using around 15% of the total photosynthetic energy [41]. As BoRHON1 can induce the accumulation of all detectable glucosinolates, to support the production of these defense compounds, plants need to increase their energy production, repress normal plant growth and development as a trade-off, or both. Our study clearly showed that BoRHON1 could specifically increase the photosynthetic rates in plants and allocate energy to glucosinolate biosynthesis while maintaining normal plant growth and development. This suggests that plant growth and plant defense are not merely a trade-off but rather a dynamic interplay [43]. BoRHON1 might be a valuable target gene for breeding and normally growing and harvesting Brassica vegetables in fields at serious risk of herbivory and biotic stresses.

## 4. Materials and Methods

### 4.1. Plant Material and Growth Conditions

*Arabidopsis thaliana* (wild type, Columbia-0) [14], cabbage (*B. oleracea* var. *capitata* line 02–12) [34], and tobacco (*Nicotiana benthamiana*) were used in this study. The cabbage and tobacco were seeded into a mixture of matrix, vermiculite, and perlite with a ratio of 1:1:1, and cultivated in a growth chamber at 25 °C, light intensity of 125 μmol·m^−2^·s^−1^, and light cycle of 16 h of light/8 h of darkness. Arabidopsis seeds were incubated in darkness at 4 °C for 48 h to ensure synchronous germination. Seeds were seeded in a mixture of matrix, vermiculite, and perlite at a ratio of 1:1:1. The seeds were cultured in an incubator with a temperature of 22 °C, light intensity of 125 μmol·m^−2^·s^−1^, and light cycle of 16 h of light/8 h of darkness.

### 4.2. Yeast One-Hybrid Assay

The yeast one-hybrid screen was conducted using the Matchmaker^®^ Gold Yeast One-Hybrid Library Screening System (Clontech, Mountain View, CA, USA) in accordance with the manufacturer’s instructions. Genomic DNA was extracted from leaves of cabbage 02–12 using CTAB method. The 2000 bp promoter regions of *BoMYB28s* were cloned between *Hin*d III and *Kpn* I sites of pAbAi vector and transformed into Y1H Gold strain. Vector insertion was detected using Matchmaker Insert Check PCR Mix 1 (TaKaRa, San Jose, CA, USA). Aureobasidin A (AbA) was used for self-activation detection. A total of 10 μg of cabbage cDNA library plasmid was transformed into p*BoMYB28-1-Pro-4*-AbAi, p*BoMYB28-2-Pro-7*-AbAi, and p*BoMYB28-3-Pro*-AbAi bait strains, respectively, and then selected on SD/-Leu medium with corresponding concentrations of AbA. After 3–4 d, the normally growing colonies were identified by PCR using the T7 primer (TAATACGACTCACTATAGGG), and bands larger than 500 bp were selected for sequencing. All primer sequences in this study are listed in Supplementary Table S6.

The encoding sequence of *BoRHON1* was inserted between the *Eco*R I and *Bam*H I sites of the pGADT7 vector. The constructed pGADT7 vector and empty vector were transformed into p*BoMYB28-3-Pro*-AbAi bait strain, respectively, and the bacteria solutions were seeded into SD/-Leu medium and SD/-Leu medium with AbA concentration of 400 ng/mL. The growth condition of yeast strains was observed after 3–4 d.

### 4.3. Transient Expression Assay in N. benthamiana Leaves

The transient expression assays were performed in *N. benthamiana* leaves. To generate the pGreenII-proBoMYB28-3:LUC reporter, the 2 kb promoter region upstream of the *BoMYB28-3* start codon was cloned using ApexHF HS DNA Polymerase CL (Accurate Biology, Changsha, China) and ligated between the *Kpn* I and *Nco* I sites of the pGreenII-0800-LUC vector using the ClonExpress II One Step Cloning Kit (Vazyme, Nanjing, China). The *BoRHON1* coding region was inserted between the *Bam*H I and *Eco*R I sites of the pGreenII-62-SK vector to generate effector. Plasmids were, respectively, transformed into *Agrobacterium tumefaciens* strain GV3101 harboring pSOUP helper plasmid. The bacterial liquid-carrying reporter or empty vector and effector was mixed according to the volume ratio of 1:9. According to the manufacturer’s instructions, dual-luciferase assays were performed using the Dual Luciferase Reporter Gene Assay Kit (YEASEN, Shanghai, China) in a full-wavelength multifunction microplate reader (Tecan, Männedorf, Switzerland). The binding ability of BoRHON1 to the promoter of *BoMYB28-3* was evaluated based on the ratio of LUC to REN. In addition, a CCD imaging apparatus (PlantView100, Guangzhou, China) was used to capture the LUC images. Tobacco leaves were coated with 100 mM of luciferin (Promega, Chūō, Tokyo) and were placed in darkness for 5 min before luminescence detection.

### 4.4. Bioinformatic Analysis

We downloaded all relevant protein sequences from the Brassicaceae Database (http://brassicadb.cn/#/, accessed on 5 November 2023). Multiple sequence alignments of RHON1s were carried out using DNAMAN software V6.0.3.40 (LynnonBiosoft, San Ramon, CA, USA). MEGA X software was used to construct the phylogenetic tree of RHON1s with the neighbor-joining (NJ) method (https://www.megasoftware.net/, accessed on 4 November 2023), bootstrap test was performed, and repeated value was set to 1000. SWISS MODEL (https://swissmodel.expasy.org/, accessed on 5 November 2023) was used to predict the protein tertiary structure of BoRHON1.

### 4.5. Transcriptional Activation Activity Assay

The coding sequence of *BoRHON1* was ligated between the *Eco*R I and *Bam*H I sites of the pGBKT7 vector, the pGBKT7 plasmid with the target gene fragment, and pGADT7 plasmid. Positive control (pgBKT7-53 + pgADT7-T) and negative control (pGBKT7 + pGADT7) were co-transferred into Y2H Gold yeast strain. The transformed yeast cells were coated on DDO (SD/-Trp-Leu) solid medium and cultured at 30 °C for 3–5 days. The yeast monoclonal colonies were selected and seeded into QDO solid medium (SD/-Trp-Leu-His-Ade) and SD/-Trp-Leu-His-Ade+x-a-Gal. The transcriptional activation activity of BoRHON1 was assessed using growth status and the color of yeast cells.

### 4.6. Subcellular Localization Analysis

The coding sequence of *BoRHON1* without the stop codon was cloned and ligated between the *Xho* I and *Eco*R I sites of the pGreen-35S:GFP vector. The pGreen-35S:BoRHON1:GFP and pGreen-35S:GFP empty vector plasmids were transformed into *Agrobacterium tumefaciens* strain GV3101 harboring pSOUP helper plasmid. When the *Agrobacterium* solution OD_600_ was 0.6, we centrifuged the bacterial solution at 4000 rpm for 10 min at 4 °C; the precipitates were re-suspended with infection solution (infection solution components: 10 mmol/L MgCl_2_, 10 mmol/L MES, adjusted pH to 5.6, acetosyringone added at a final concentration of 100 μmol/L) and then placed at room temperature for 2 h. The bacterial solution was injected into the back of tobacco leaves using a 1 mL syringe. The distribution of fluorescent proteins in the cells of the injected tobacco was observed 2 days later using Laser Scanning Confocal Microscope (Leica, Wetzlar, Germany).

### 4.7. Quantitative Real-Time RT-PCR Analysis

Total RNA from different tissues of cabbage (roots, stems, leaves, flowers, and siliques) and rosette leaves of *A. thaliana* were extracted using the RNAprep Pure Plant kit (Tiangen, Beijing, China), and 1 μg of RNA was used for reverse transcription using HiScript III 1st Strand cDNA Synthesis Kit (+gDNA wiper) (Vazyme, Nanjing, China). Quantitative real-time PCR (qRT-PCR) analysis was performed by QuantStudio^®^3 (Life Technologies, Carlsbad, CA, USA) using Hieff^®^ qPCR SYBR Green Master Mix (Low Rox Plus) (YEASEN, Shanghai, China) with three biological replicates and technical replicates. The cabbage actin gene (*BoACTIN*, BolC01g044090.2J) and Arabidopsis actin gene (*AtACT2*, AT3G18780) were used as controls. The relative gene expression was calculated using the 2^−ΔΔCT^ method.

### 4.8. Generation of Transgenic Arabidopsis Plants

The encoding sequence of *BoRHON1* was inserted between the *Xba* I and *Kpn* I sites of pVBG-2307 vector [44], and pVBG2307-BoRHON1 was constructed and expressed in Arabidopsis via *Agrobacterium tumefaciens* strain GV3101. The transgenic seeds were cultured on Murashige and Skoog (MS) medium with kanamycin, and PCR was used to detect vector insertion. The positive strains of T_3_ generation were detected by qRT-PCR. *AtACT2* (AT3G18780) was used as the internal reference gene. Then, 3-week-old unbolted seedlings of wild type (WT) and T_3_ generations of homozygous lines (OE-1, OE-2) were used for the experiments.

### 4.9. Glucosinolate Extraction and Analysis

The collection procedure of plant leaf samples used for GLS was similar to that described previously but appropriately modified to fit the HPLC platform used in this study [45]. Briefly, 2–3 fully mature leaves were removed from each 3-week-old plant, placed in 1000 μL of 90% (*v*/*v*) methanol, and stored at −80 °C before extraction. Samples were broken using a 2.3 mm metal ball bearing in a paint shaker at room temperature and incubated for 1 h at room temperature. The tissues were centrifuged at 2500× *g* for 15 min, and the supernatant was subjected to anion exchange chromatography in 2 mL tubes. After methanol and water washing, the columns were incubated with 210 μL sulfatase solution overnight. The desulfo-GLS were eluted and analyzed by HPLC according to previously described method.

### 4.10. Determination of Photosynthetic Parameters

The 3-week-old unbolted seedlings of wild type (Col-0) and T_3_ generation *BoRHON1* Arabidopsis overexpression lines (OE-1, OE-2) were used to determine photosynthetic parameters. The net photosynthetic rate (A), transpiration rate (E), intercellular CO_2_ concentration (Ci), and stomatal conductance to water (gsw) of mature rosette leaves of Arabidopsis were measured using the plant photosynthetic meter (LI-6800, USA). All values were calculated from three biological replicates.

### 4.11. Statistical Analysis

Statistical analysis was performed using SPSS 23.0 software (IBM Inc., Chicago, IL, USA). Statistical significance was determined using Student’s *t*-test. All data are presented as the means ± SE (standard error). Treatments were considered significantly different at *p* ≤ 0.05.

## 5. Conclusions

The upstream regulators of three *BoMYB28s* in cabbage were systemically screened and identified using a yeast one-hybrid assay. BoRHON1, an RNA-binding protein, was selected and validated as a strong positive regulator of both aliphatic and indolic glucosinolates, as it was able to induce the accumulation of every detectable glucosinolate. Importantly, BoRHON1 could increase the photosynthetic rate to specifically boost glucosinolate biosynthesis, despite the high energy costs of glucosinolates, while maintaining the normal growth and development of the plants.

## Data Availability

All the data supporting the findings of this study are available within the paper and in its Supplementary Materials, published online. The plant materials used in this study are available upon request.

## Supplementary Materials

The following supporting information can be downloaded at: https://www.mdpi.com/article/10.3390/ijms25105314/s1.
