# Supplementary material for: The RNA-Binding Protein BoRHON1 Positively Regulates the Accumulation of Aliphatic Glucosinolates in Cabbage
Source: Int J Mol Sci. 2024 May 13;25(10):5314. doi: 10.3390/ijms25105314 (PMC11120748; doi:10.3390/ijms25105314)
Supplement: Supplementary file 1 [file ijms-25-05314-s001.zip › Figures S1-S7.pptx]

## Slide 1
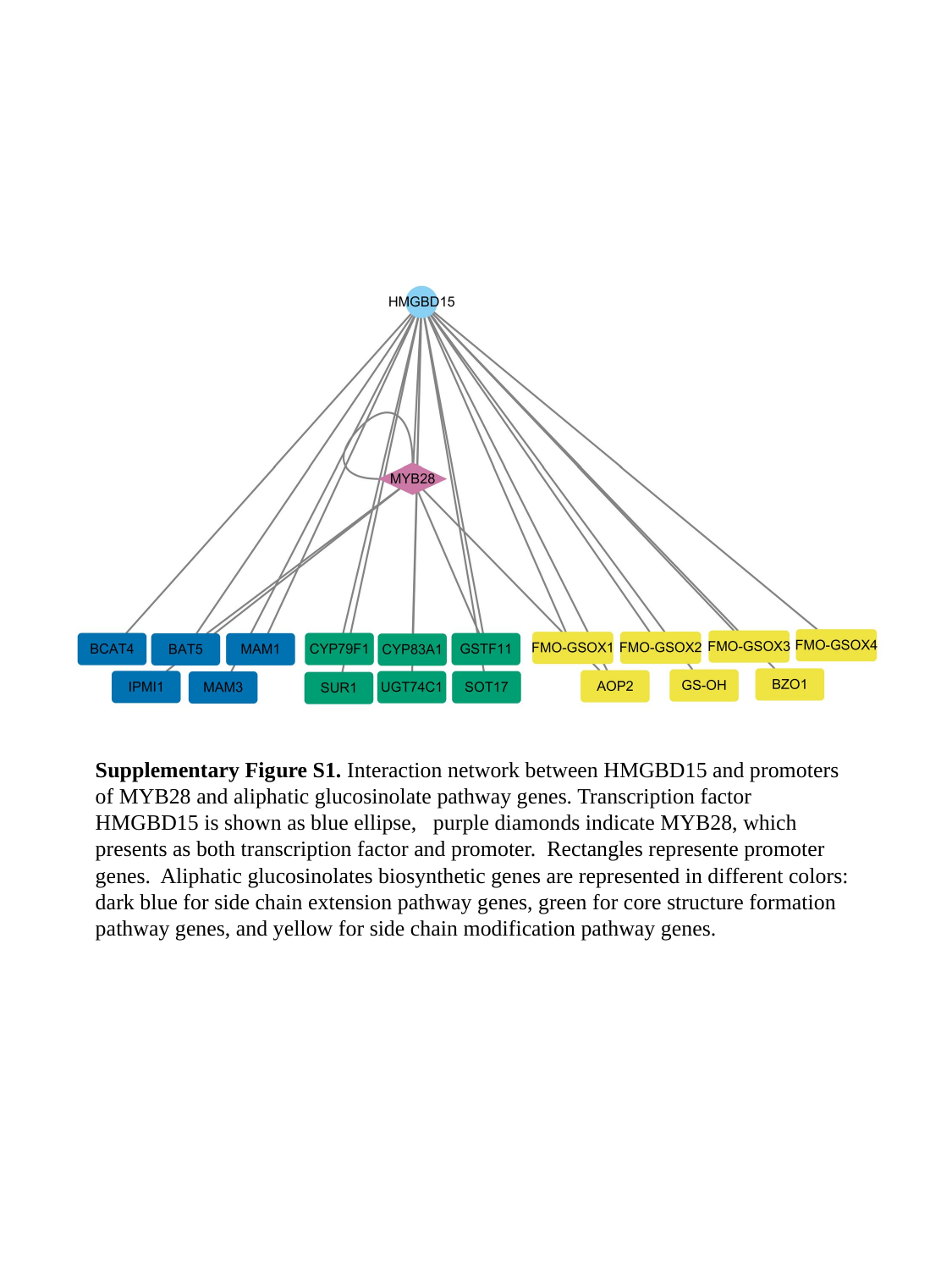

Supplementary Figure S1. Interaction network between HMGBD15 and promoters of MYB28 and aliphatic glucosinolate pathway genes. Transcription factor HMGBD15 is shown as blue ellipse, purple diamonds indicate MYB28, which presents as both transcription factor and promoter. Rectangles represente promoter genes. Aliphatic glucosinolates biosynthetic genes are represented in different colors: dark blue for side chain extension pathway genes, green for core structure formation pathway genes, and yellow for side chain modification pathway genes.

## Slide 2
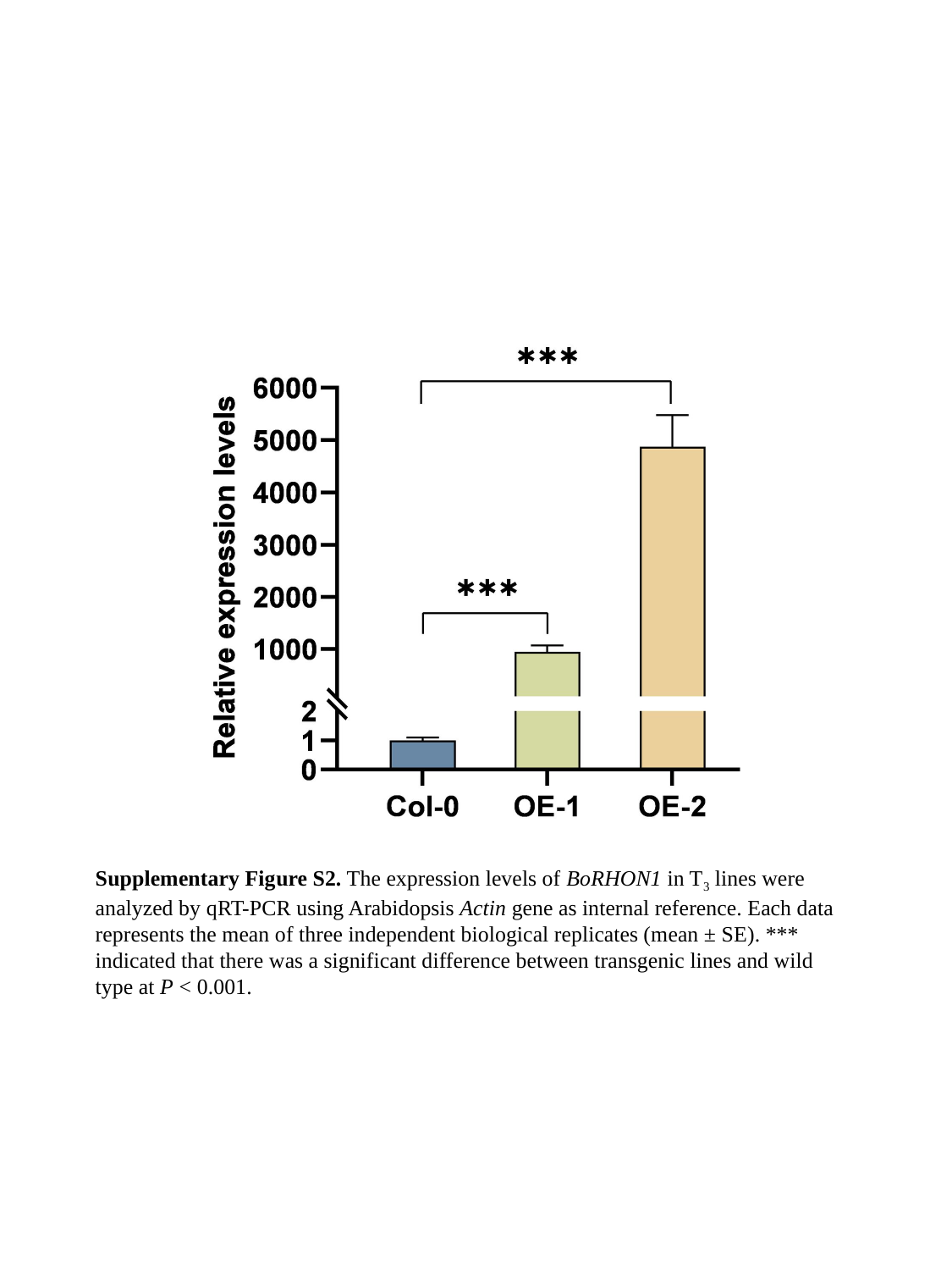

Supplementary Figure S2. The expression levels of BoRHON1 in T3 lines were analyzed by qRT-PCR using Arabidopsis Actin gene as internal reference. Each data represents the mean of three independent biological replicates (mean ± SE). *** indicated that there was a significant difference between transgenic lines and wild type at P < 0.001.

## Slide 3
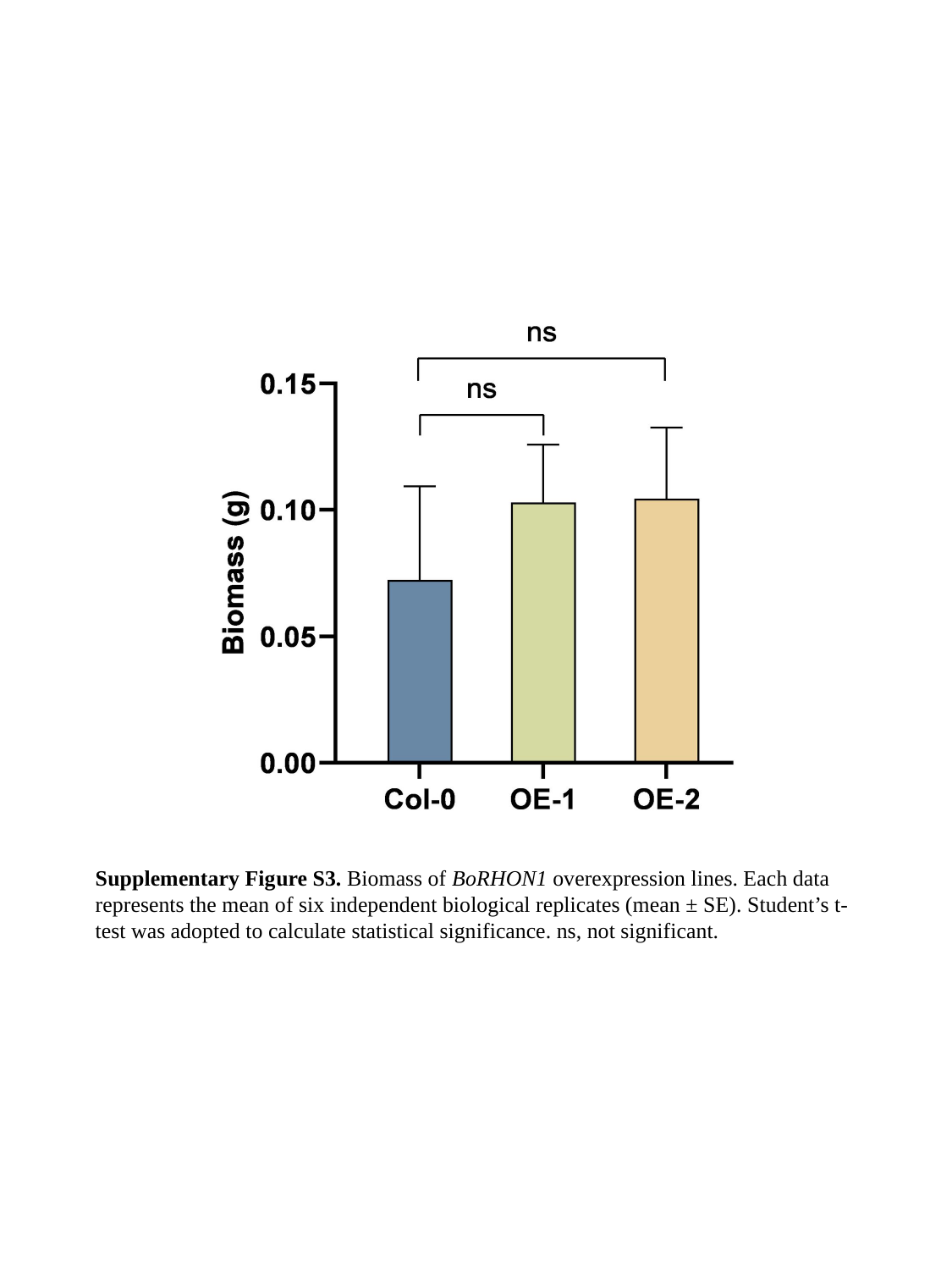

Supplementary Figure S3. Biomass of BoRHON1 overexpression lines. Each data represents the mean of six independent biological replicates (mean ± SE). Student’s t-test was adopted to calculate statistical significance. ns, not significant.

## Slide 4
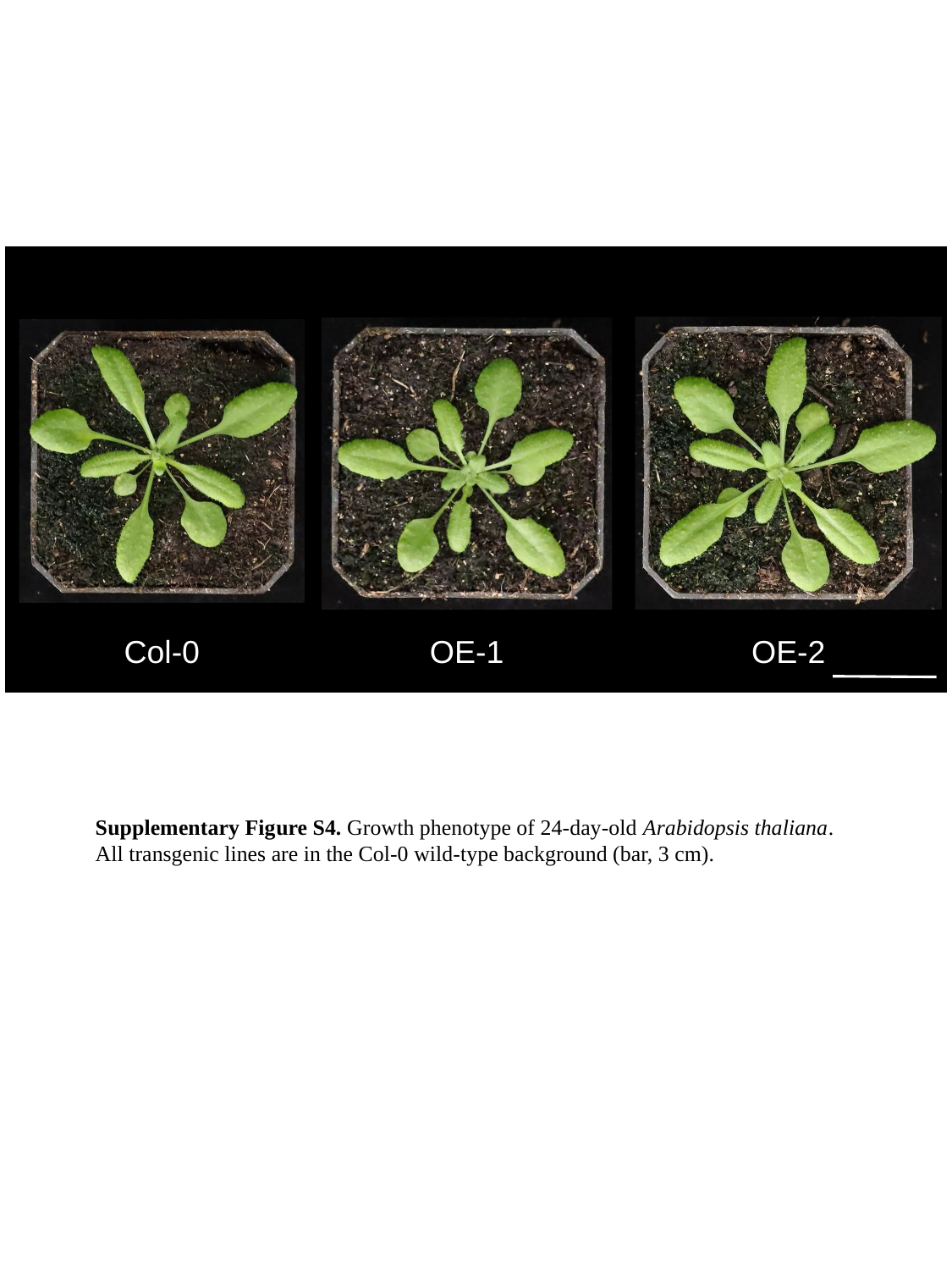

Col-0
OE-1
OE-2
Supplementary Figure S4. Growth phenotype of 24-day-old Arabidopsis thaliana. All transgenic lines are in the Col-0 wild-type background (bar, 3 cm).

## Slide 5
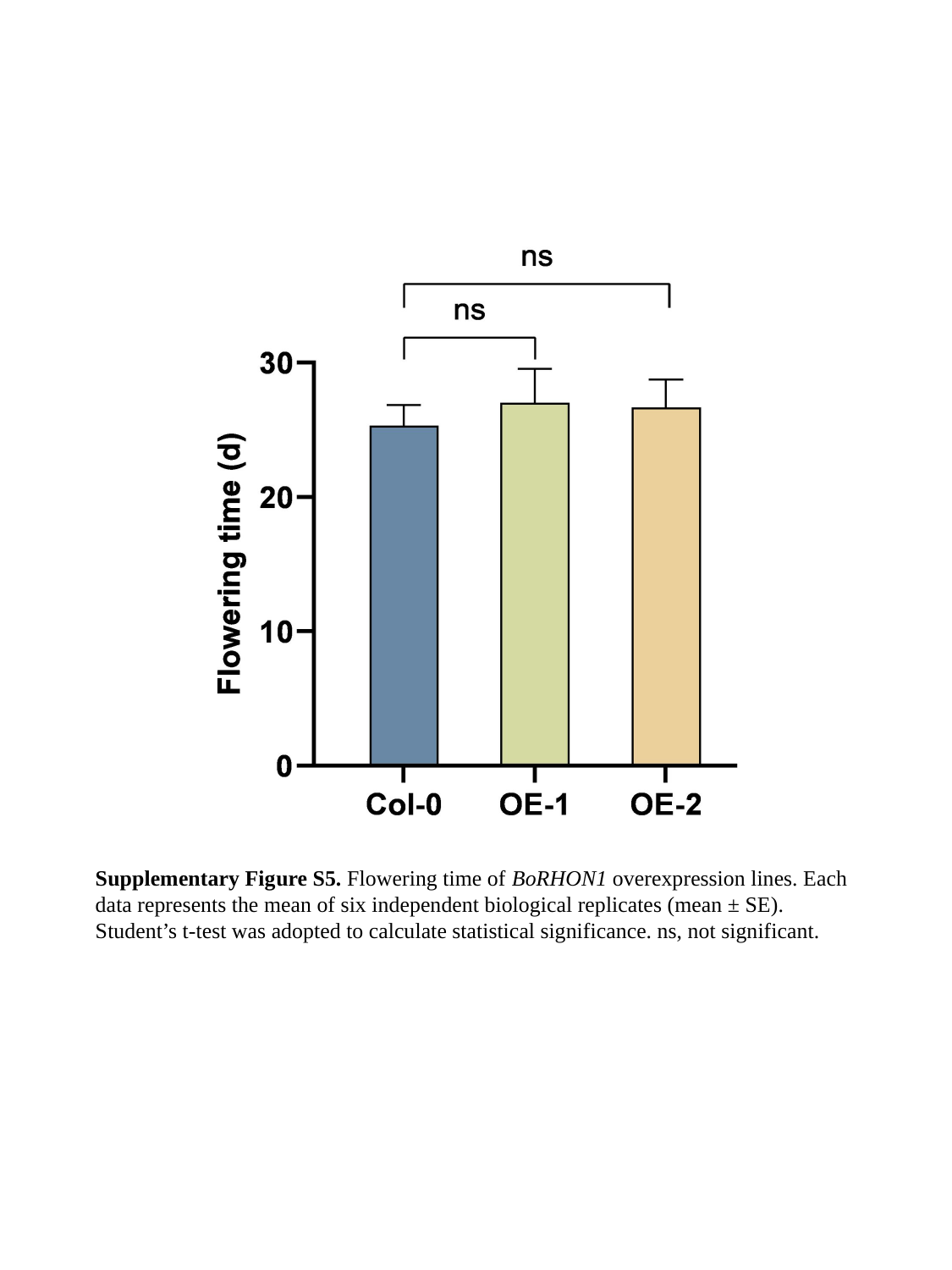

Supplementary Figure S5. Flowering time of BoRHON1 overexpression lines. Each data represents the mean of six independent biological replicates (mean ± SE). Student’s t-test was adopted to calculate statistical significance. ns, not significant.

## Slide 6
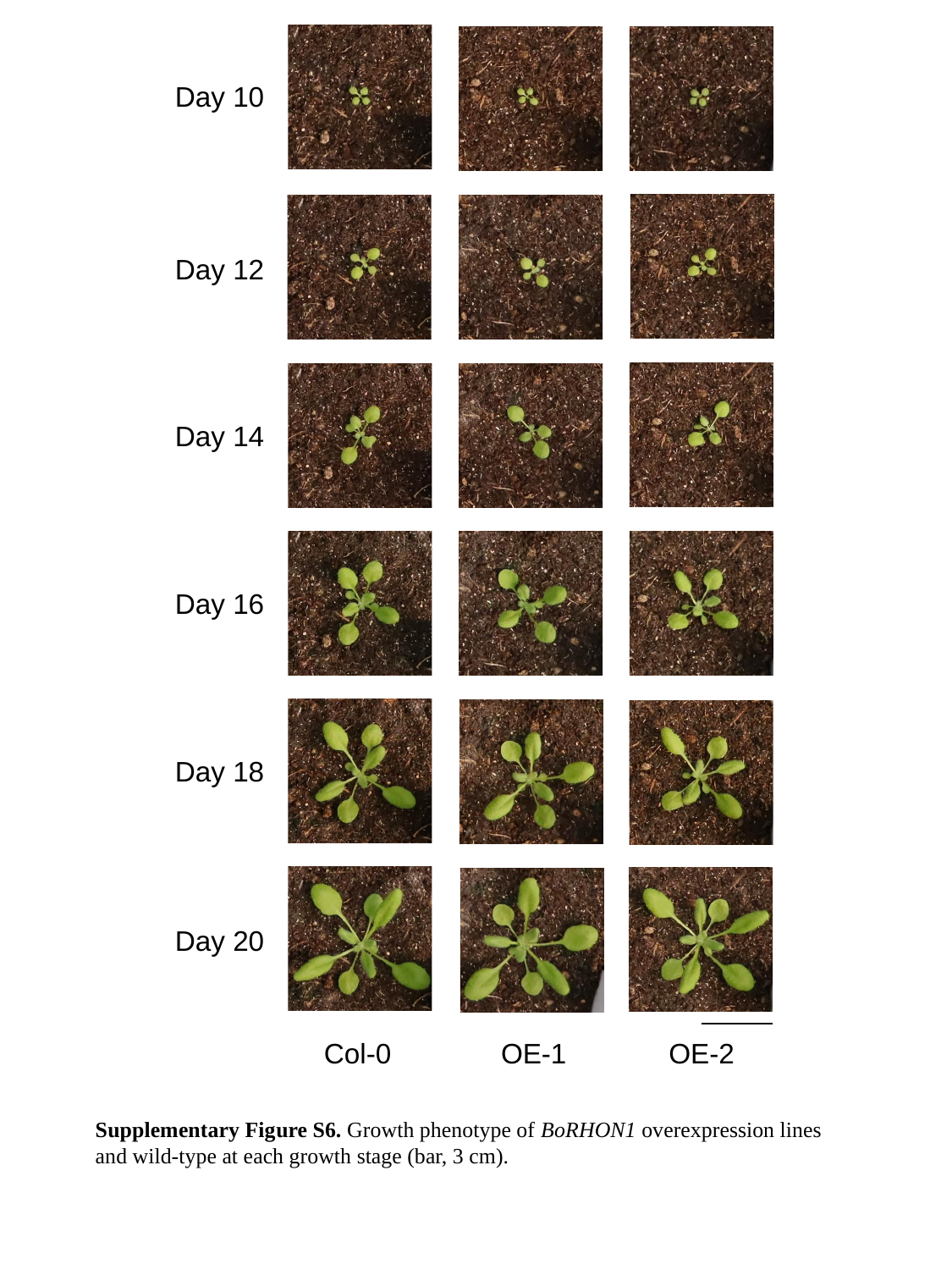

Day 10
Day 12
Day 14
Day 16
Day 18
Day 20
Col-0
OE-1
OE-2
Supplementary Figure S6. Growth phenotype of BoRHON1 overexpression lines and wild-type at each growth stage (bar, 3 cm).

## Slide 7
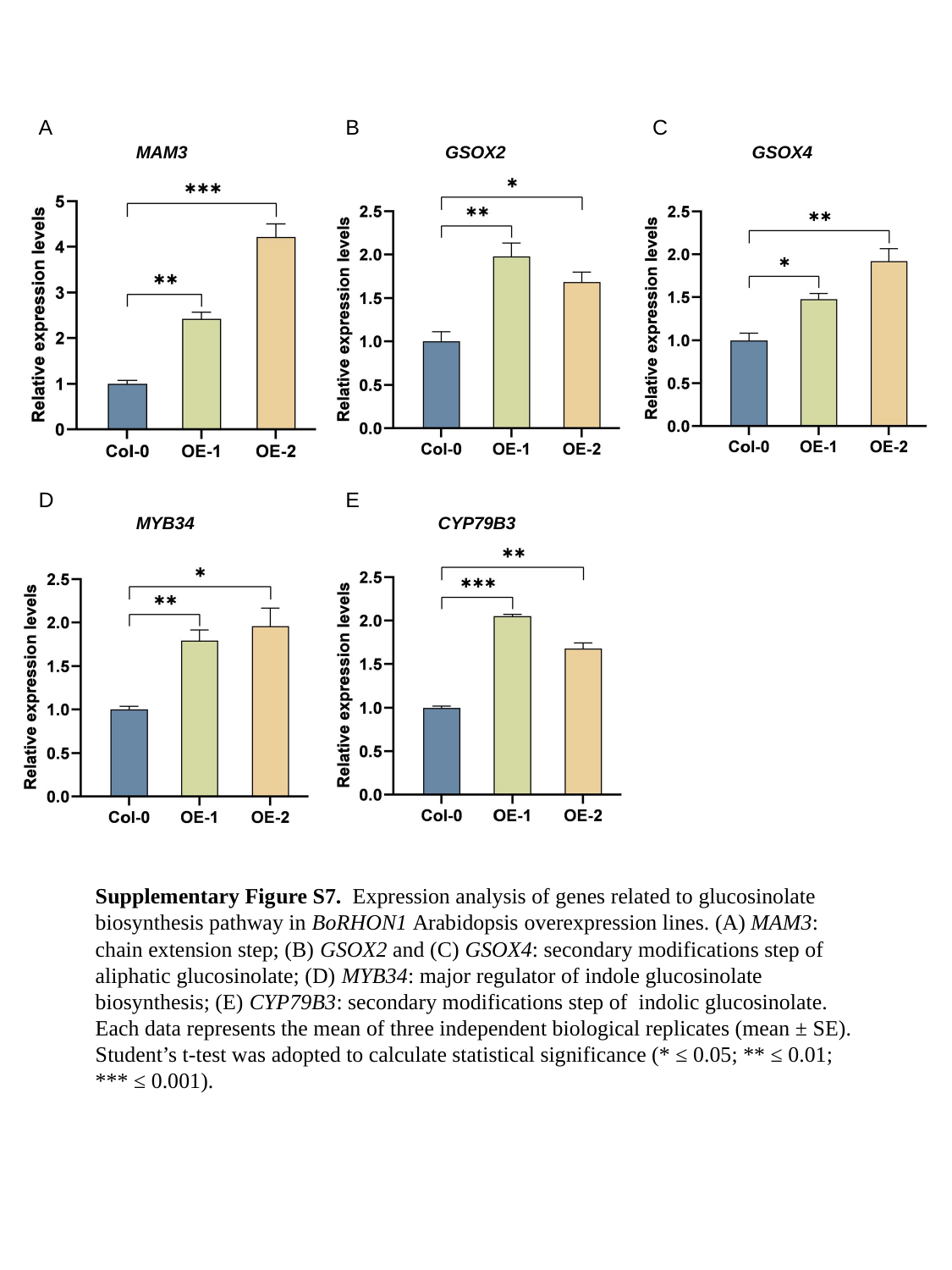

A
B
C
MAM3
GSOX2
GSOX4
D
E
MYB34
CYP79B3
Supplementary Figure S7. Expression analysis of genes related to glucosinolate biosynthesis pathway in BoRHON1 Arabidopsis overexpression lines. (A) MAM3: chain extension step; (B) GSOX2 and (C) GSOX4: secondary modifications step of aliphatic glucosinolate; (D) MYB34: major regulator of indole glucosinolate biosynthesis; (E) CYP79B3: secondary modifications step of indolic glucosinolate. Each data represents the mean of three independent biological replicates (mean ± SE). Student’s t-test was adopted to calculate statistical significance (* ≤ 0.05; ** ≤ 0.01; *** ≤ 0.001).
